# Analysis of DNA Methylation Differences during the JIII Formation of *Bursaphelenchus xylophilus*

**DOI:** 10.3390/cimb45120603

**Published:** 2023-11-30

**Authors:** Peng Wang, Yongxia Li, Zhenkai Liu, Wei Zhang, Dongzhen Li, Xuan Wang, Xiaojian Wen, Yuqian Feng, Xingyao Zhang

**Affiliations:** 1 Key Laboratory of Forest Protection of National Forestry and Grassland Administration, Ecology and Nature Conservation Institute, Chinese Academy of Forestry, Beijing 100091, China; wangpeng9811@163.com (P.W.); oklzkk@163.com (Z.L.); zhangwei1@caf.ac.cn (W.Z.); lidongzhen1949@163.com (D.L.); jiuwozhidao@163.com (X.W.); wenxj1016@126.com (X.W.); fengyuqian1988@163.com (Y.F.); zhangxingyao@126.com (X.Z.); 2 Co-Innovation Center for Sustainable Forestry in Southern China, Nanjing Forestry University, Nanjing 210037, China

**Keywords:** whole genome bisulfite sequencing, DNA methylation, *Bursaphelenchus xylophilus*, differentially methylated regions, gene expression, environmental suitability

## Abstract

DNA methylation is a pivotal process that regulates gene expression and facilitates rapid adaptation to challenging environments. The pinewood nematode (PWN; *Bursaphelenchus xylophilus*), the causative agent of pine wilt disease, survives at low temperatures through third-stage dispersal juvenile, making it a major pathogen for pines in Asia. To comprehend the impact of DNA methylation on the formation and environmental adaptation of third-stage dispersal juvenile, we conducted whole-genome bisulfite sequencing and transcriptional sequencing on both the third-stage dispersal juvenile and three other propagative juvenile stages of PWN. Our findings revealed that the average methylation rate of cytosine in the samples ranged from 0.89% to 0.99%. Moreover, we observed significant DNA methylation changes in the third-stage dispersal juvenile and the second-stage propagative juvenile of PWN, including differentially methylated cytosine (DMCs, *n* = 435) and regions (DMRs, *n* = 72). In the joint analysis of methylation-associated transcription, we observed that 23 genes exhibited overlap between differentially methylated regions and differential gene expression during the formation of the third-stage dispersal juvenile of PWN. Further functional analysis of these genes revealed enrichment in processes related to lipid metabolism and fatty acid synthesis. These findings emphasize the significance of DNA methylation in the development of third-stage dispersal juvenile of PWN, as it regulates transcription to enhance the probability of rapid expansion in PWN.

## 1. Introduction

**Figure 1:**
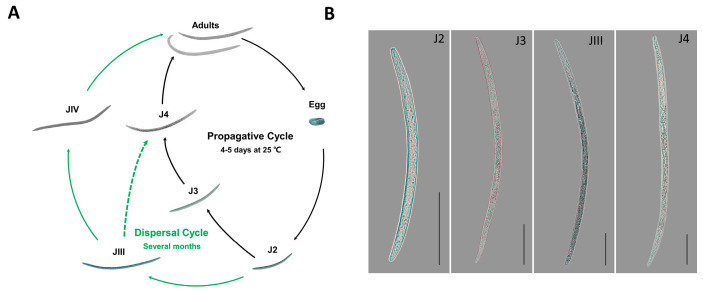
Life cycle of pine wood nematode and morphology of tested nematodes. (**A**) Propagative cycle and dispersal cycle of PWN. J2, second–stage propagative juvenile; J3, third–stage propagative juvenile; J4, fourth–stage propagative juvenile; JIII, third–stage dispersal juvenile; JIV, fourth–stage dispersal juvenile. (**B**) PWN at different developmental stages was used in this experiment. The scale indicates 100 μm.

The pine wood nematode (PWN; *Bursaphelenchus xylophilus*) is a devastating pathogen that causes pine wilt disease in pine trees, and pine trees infected with PWN will eventually die [1,2]. This microscopic nematode has a complex life cycle, with developmental stages of phytophagy (feeding on plants) and mycetophagy (feeding on fungi), as well as different life cycles, including propagative and dispersal cycles (Figure 1A) [3]. After being transmitted into the living tree through the vector insect (*Monochamus* spp.), the PWN initially feeds on the epithelial cells surrounding the cortex and xylem, and then migrates within the tree primarily through resin canals (phytophagous stage). As the tree starts to exhibit signs of withering and other symptoms, the PWN switches to feeding on the fungi present inside the tree (mycophagia). During this process, PWN undergoes a propagative cycle, starting with the hatching of eggs into three propagative juvenile stages (J2, J3, and J4), which eventually develop into adults [1,4,5]. When autumn arrives, unfavorable living conditions such as low temperatures, dryness, and food scarcity start to appear. This triggers a halt in the reproductive cycle of PWN, and the second-stage propagative juvenile (J2) gradually develop into the third-stage dispersal juvenile (JIII) [6,7]. JIII is capable of withstanding adverse external conditions, and under the influence of the vector insect, *Monochamus* beetles, transforms into the fourth-stage dispersal juvenile (JIV) in the following spring. When the *Monochamus* beetles emerge and feed on healthy pine trees, JIV is transferred to the trees. Under suitable environmental conditions, JIV transforms and develops into adults within the pine trees, thus entering the reproductive cycle [8,9]. Therefore, understanding the formation mechanism of JIII is crucial for preventing the spread of PWN.

Numerous studies have shown that external environmental stress can induce epigenetic changes, including alterations in DNA methylation [10]. This reversible change allows organisms to respond rapidly to environmental fluctuations, thereby enhancing their adaptability to environmental stress [11,12]. DNA methylation is capable of regulating the expression of specific genes without altering the genome itself [13]. This epigenetic modification plays a crucial role in facilitating the rapid adaptation of organisms to their environment, particularly in harsh or challenging conditions [14,15]. For instance, alterations in methylation patterns triggered by temperature stress can exert a regulatory influence on the sex determination of organisms. In response to temperature induction, the European sea bass can modify the methylation level of the *cypl9a* promoter in its gonads, thereby affecting the sex ratio [16]. Similarly, sea turtles can regulate the expression of the sex-related gene *Sox9*, which enables the development of bidirectional gonads into either ovaries or testes [17]. DNA methylation can affect the differentiation of honeybee queens and worker bees and the rapid response of zebrafish to external environmental stress, thereby improving the adaptability of the population to adverse environments [18,19,20]. Although the aforementioned studies have provided evidence that environmental changes can alter the DNA methylation patterns of organisms, there is a dearth of research on whether DNA methylation modifications occur in PWN, enabling JIII to swiftly adapt to environmental changes.

DNA methylation is a crucial regulatory mechanism in the epigenetic process. DNA methylation is a process in which DNA methyltransferases (DNMTs) catalyze the transfer of the methyl group from the methyl donor S-adenosylmethionine (SAM) to specific bases in DNA [21]. 5-methylcytosine (5mC) is a prominent DNA modification found in eukaryotes, and its methylase is conserved across various biological species, including animals, plants, and fungi [22]. In most vertebrate cells, approximately 60–90% of CpG dinucleotides are modified to 5-methylcytosine (5mC), which is believed to contribute to genome stability [23,24]. In contrast, the degree of DNA methylation varies greatly in invertebrate genomes, with a tendency towards sparse methylation. In these genomes, DNA methylation is primarily targeted to specific transcriptional units, rather than being widespread [25].

In the study of DNA methylation modifications in nematodes, significant differences were observed in the level and pattern of DNA methylation among nematode species, and these landscapes also exhibit dynamic changes over different periods [22,26]. As a model organism, *Caenorhabditis elegans* showed a complete absence of cytosine methylation but exhibited low-level adenine methylation modifications [27]. Conversely, cytosine methylation was found in the animal parasitic nematode *Trichinella spiralis*, confirming the presence of DNA cytosine methylation in nematodes [28]. In the distribution of DNA cytosine methylases among the major groups of nematodes, it was discovered that the enzymes necessary for 5mC methylation were either partially or completely absent in certain nematode groups [22]. However, the precise level and pattern of DNA methylation in PWN, particularly across its entire genome, remain uncertain.

Changes in DNA methylation often lead to changes in gene expression, making the joint analysis of DNA methylation and transcriptome crucial in the thorough investigation of biological growth and environmental adaptation [29,30]. Bisulfite sequencing is currently one of the most accurate and effective methods for obtaining single-base resolution biological DNA methylation profiles, enabling the description of genome-wide DNA methylation profiles [31]. In this study, we performed whole-genome bisulfite sequencing (WGBS) on four different developmental stages in PWN, constructing a comprehensive methylation map of its genome. We analyzed the differences in genomic methylation during JIII formation to gain insights into the role of DNA methylation in environmental adaptation, as well as to identify DNA methylation modification signatures associated with fitness. In addition, we employed RNA-seq to integrate methylation changes and differences in gene expression, enabling us to identify differentially methylated regions and associated genes during JIII formation to elucidate the regulatory role of DNA methylation in the environmental adaptation of PWN.

## 2. Materials and Methods

### 2.1. Nematodes in Different Developmental Stages

Different developmental stages of PWNs were obtained from Fushun City (Liaoning Province, China) in October 2022. The PWNs in the form of JIII were selected in deionized water. The JIII were cultured on the mycelia of *Botrytis cinerea* on potato dextrose agar (PDA) (Becton, Dickinson and Company, Sparks, MD, USA) plates at 25 °C for 5 days incubator without light to obtain different stages of propagative juvenile. Eggs of the PWNs were collected in glass dishes and incubated at 25 °C in the dark for 24 h, then almost all the eggs hatched to J2. The J2 were inoculated on *B. cinerea* and cultured for 30 h and 54 h to obtain J3 and J4 in turn.

### 2.2. DNA and RNA Extraction

Genomic DNA for 12 groups of PWNs from different stages (3 replicates for each stage: J2, J3, JIII, J4) was extracted using the DNeasy Blood and Tissue kit from Qiagen. The DNA was extracted following the Spin-column Protocol for Purification of Total DNA from Animal Blood or Cells (Qiagen, Venlo, The Netherlands, July 2020). DNA was quantified using the Qubit dsDNA HS Assay Kits for fluorometric quantification on a Qubit 4 Fluorometer (Thermo Fisher Scientific, Waltham, MA, USA). The DNA extract was used for both WGBS and subsequent validation analysis.

Total RNA was extracted separately from the powders of different stages of PWNs using the TransZol Up Plus RNA Kit (TransGen Biotech, Beijing, China). RNA was quantified using the Qubit RNA BR Assay Kits for fluorometric quantification on a Qubit 4 Fluorometer (Thermo Fisher Scientific). RNA degradation and contamination were monitored on 1% agarose gel electrophoresis. The RNA extract was used for both RNA-seq and subsequent qRT-PCR.

### 2.3. WGBS Library Preparation

Before constructing the library, the Bioruptor system was utilized to fragment a mixture of 1 µg of genomic DNA and unmethylated lambda DNA resulting in an average size of approximately 250 bp. After the DNA is fragmented, it undergoes a process of repair, blunting, and phosphorylation of the ends with a mixture of T4 DNA polymerase (Thermo Fisher Scientific), Klenow fragment, and T4 polynucleotide kinase. The blunt DNA fragments are first subjected to 3′-adenylation using the Klenow fragment, which targets the 3′-5′ exons. The resulting fragments are then ligated to adapters containing 5′-methylcytosines instead of regular cytosines, using T4 DNA ligase. After each step, DNA was purified using the QIAquick PCR purification kit (Qiagen). Unmethylated cytosines were then converted to uracil using the ZYMO EZ DNA Methylation-Gold Kit™ (Zymo Research, Orange County, CA, USA) according to the instructions. This kit involves treating methylated DNA with bisulfite, which converts unmethylated cytosines into uracil. Methylated cytosines remain unchanged during the treatment. Once converted, the methylation profile of the DNA can be determined by PCR amplification followed by DNA sequencing. Adding lambda sequences during library construction allows for the evaluation of the library’s transformation efficiency. Lambda sequences are DNA sequences that are completely unmethylated. The actual conversion efficiency of the library can be determined by calculating the ratio of C to T conversion in the lambda sequence, which represents the methylation rate. Finally, perform PCR in a final reaction volume of 50 µL containing 20 µL ligated DNA, 4 µL 2.5 mM dNTPs, 5 µL 10× buffer, 0.5 µL JumpStart™ Taq DNA polymerase, 2 µL PCR primers, and 18.5 µL water as per thermocycling program, at 94 °C for 1 min, 94 °C for 10 s, 62 °C for 30 s, 72 °C for 30 cycles, then 72 °C for 5 min extension, the product can be stored at 12 °C. The PCR products were purified using a QIAquick PCR purification kit (Qiagen). Before analysis with the Illumina sequencing platform, the purified library was analyzed by the Bioanalyzer analysis system (Agilent, Santa Clara, CA, USA) and quantified by real-time PCR.

### 2.4. Sequence Alignment and Detection of Cytosines Methylation Level

Low-quality bases and adapter sequences were trimmed by using Trimmomatic (v0.38). The parameter settings used were “SLIDINGWINDOW:5:15 HEADCROP:3 AVGQUAL:15 LEADING:5 TRAILING:5 MINLEN:80”. The filtering process includes removing the adapter sequence, discarding reads with an average base quality value below 15, trimming bases with a quality value less than 5 from both ends, and removing sequences shorter than 80 bp. The cleaned reads were mapped back to the reference genome (BioSample: SAMEA7232845, https://www.ncbi.nlm.nih.gov/assembly/GCA_904066235.2 (accessed on 5 December 2022)) [32] using BSMAP software version 2.90. The parameter settings used were “-n 0 -v 0.08 -g 1 -p 48”. Methylation ratios were extracted from BSMAP output (SAM) using a Python script (methratio.py) which is distributed with the BSMAP package. In brief, the methylation level was calculated based on the methylated cytosine (mC) percentage in the whole genome as site methylation level = 100 × (number of sequences with methylated cytosines (mC)/total number of valid sequences).

### 2.5. DMC and DMR Detection

Differentially methylated cytosines (DMCs) were detected using MethylKit (v1.20) in de novo mode, considering CpG sites with a minimum coverage of 5×. The parameter settings used were “--mincov 5 --m_cutoff 0.1 --q_cutoff 0.05”. Differentially methylated regions (DMRs) were detected using Metilene in de novo mode among CpG sites with at least 5× coverage. The parameter settings used were “–mincpgs 1 –minMethDiff 0.1 –mode 1 -mtc 2”. The detected DMRs were filtered according to the following criteria: (1) corrected MWU-test *p*-value less than 0.05; (2) methylation level difference greater than 0.1; (3) DMR must contain more than 1 CpG site; and (4) DMR length must be greater than 10 bp. The genomic locations of DMCs and DMRs were determined using the genome GFF file. The genome GO and KEGG annotation was performed using emapper based on the EGGNOG database. The identified genes were further subjected to GO and KEGG enrichment analysis using Allenricher (v1.0) [33].

### 2.6. RNA Sequencing

Purification of mRNA containing poly(A) from about 1 μg of total RNA using Oligo(dT) Beads. The captured mRNA was first fragmented into 100–200 nt using divalent cations at elevated temperatures. The fragmented mRNA was reverse transcripted with SuperScript II and then converted to double-stranded cDNA using RNaseH and DNA Pol I by random priming. After purification, the double-stranded cDNA was subjected to the blunt ending, dA addition to 3′-end, and adapter ligation. Finally, PCR was carried out to enrich the adapter-ligated cDNA, and the libraries were analyzed by Agilent Bioanalyzer 2100 and quantified by qPCR before being sequenced by the Illumina sequencing platform.

The RNA-seq libraries were constructed by E-GENE Co., Ltd. as follows. Quality control of raw reads was performed using FastQC (version 0.11.5) (https://www.bioinformatics.babraham.ac.uk/projects/fastqc/ (accessed on 20 December 2022)), and clean reads were obtained following the removal of low-quality reads with Trimmomatic (version 0.38) software [34]. The parameter settings used were “HEADCROP:8 LEADING:3 TRAILING:3 SLIDINGWINDOW:4:15 MINLEN:36”. Hisat2 (v2.2.1) [35] was employed for performing read mapping to *B. xylophilus* genome reference (v1.6.2). Gene expression profiling was based on the number of reads. TPM (Transcripts Per Kilobase of exon model per million mapped reads) values were calculated by StringTie (v2.1.7) [36], and were used to estimate the expressed values. DEGs were obtained using DESeq2 (v1.34.0) [37] with a padj value cutoff < 0.05 and an absolute log2(fold change) of >1. KEGG and GO enrichment analysis was performed on the clusterProfiler R package (v4.4.2) [38]. The GO enrichment cutoff was a q-value of <0.05, and the KEGG enrichment cutoff was a *p*-value of <0.05.

### 2.7. Validation of RT-qPCR

To assess the reliability of WGBS, three DNA fragments containing -CCGG- restriction sites were randomly chosen for DNA methylation validation experiments. The EpiJET 5-hmC and 5-mC Analysis Kit (Thermo Scientific) was selected to evaluate two cytosine modifications, namely 5-hydroxymethylcytosine (5hmC) and 5-methylcytosine (5mC), on specific DNA fragments. The gDNA underwent glycosylation and restriction endonuclease treatment as per the instructions provided in the user manual. The processed DNA samples were then quantified for different types of cytosines using RT-qPCR. The kit includes unmethylated and fully methylated sequence fragments that serve as controls and standards. To validate the results of transcriptome analysis, a total of six genes were randomly chosen for RT-qPCR analysis on the tested PWN. cDNA was synthesized from 1000 ng of total RNA from each sample using a PrimeScript™ RT reagent Kit (TaKaRa, Beijing, China). The qPCR reactions contained 2 µL of processed DNA samples or cDNA, 0.4 µL of forward and reverse primers, 10 µL of TB Green Premix Ex Taq, and 7.2 µL of nuclease-free water as per thermocycling program at 95 °C for 30 s, for 1 cycle, 95 °C for 5 s, 60 °C for 30 s, for 40 cycles. Gene expression levels were analyzed using the Light Cycler 480 System. The primers used in this study were designed using the online software Primer-BLAST from NCBI (https://www.ncbi.nlm.nih.gov/tools/primer-blast/index.cgi?LINK_LOC=BlastHome (accessed on 5 February 2023)). All primers were synthesized commercially by BGI (Beijing, China). All primers are shown in (Table S1). The experiment was performed with 3 biological replicates and 4 technical replicates. The relative expression of genes was quantified using the 2^−ΔΔCT^ method based on cycle threshold (Ct) and dissolution profiles, with efficiency correction normalized to *β-actin* [39]. It was determined by RT-qPCR that the relative expression levels of *β-actin* in the test nematodes at four stages were consistently maintained.

## 3. Results

### 3.1. DNA Methylation Sequencing Data

To investigate the DNA methylation profile in PWN, we performed bisulfite sequencing on four groups of PWN at different developmental stages (Figure 1), each sample containing an average of 31.65 million (M) raw reads with 4.7 Gb (Table S2). After raw data filtering, which generated 31.60 M clean reads with 4.61 Gb (>Q30) (92.42%) for J2, 31.85 M clean reads with 4.66 Gb (>Q30) (92.41%) for J3, 28.77 M clean reads with 4.20 Gb (>Q30) (92.25%) for JIII, and 31.90 M clean reads with 4.66 Gb (>Q30) (92.49%) for J4, respectively, were integrated for methylation mapping and DNA methylome analysis (Table S2).

### 3.2. Distribution and Statistics of Cytosine Methylation

**Figure 2:**
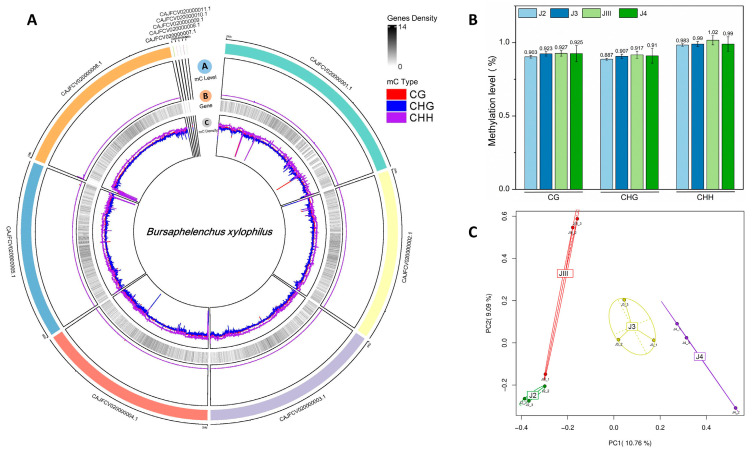
Global patterns of DNA methylation. (**A**) Whole genome methylation profile of eleven scaffolds of *Bursaphelenchus xylophilus*. (Ⓐ) mC level. (Ⓑ) Gene density. (Ⓒ) mC density. (**B**) Average methylation levels of four life stages. (**C**) PCA analysis of four life stages.

The methylation data of four groups of nematode samples at different developmental stages were compared with the reference genome. Approximately 6–28 M clean reads were mapped to the reference genome of PWN (Table S2). To enhance the content of the reference genome of PWN and improve the intuitiveness of DNA methylation analysis, we integrated methylation sequencing data into the genome. This integration allowed us to obtain the whole genome methylation profile of *B. xylophilus* (Figure 2A). According to the distribution of cytosine methylation on the genome of PWN, more than 90% of cytosine has a methylation level below 10%. The overall cytosine methylation rate in the entire genome remains consistently low (Figure 2A). After comparing the results and analyzing the methylation site information, we found that among the 12 nematode samples tested, the total cytosine methylation level ranged from 0.89% to 0.99%, except for J4_2 (Table S3). The average level of methylation in J4_2 was significantly higher compared to other nematode samples in the J4 group. However, the number of cytosine methylation sites was similar. This inconformity was attributed to the low proportion of genome mapping. Therefore, only the statistics of PWN of different developmental stages were considered, excluding J4_2 from the overall average in the analysis. Consistent with other organisms with cytosine methylation modifications, DNA cytosine methylation in PWN occurs in the context of three sequences: CG, CHG, and CHH. In the four different developmental stages of PWN, there were no significant differences in the proportions of the three sequence backgrounds of cytosine methylation. The methylation levels ranged between 0.89% and 1.02% (Figure 2B). The JIII group had a slightly higher methylation level compared to the other three groups (Figure 2B). We investigated the methylation patterns in various genomic elements, such as the average methylation rate of up2kb, genebody, and down2kb methylation sites for each transcript. In addition, we conducted a PCA analysis and observed distinct methylation differences between the different stages of our study (Figure 2C). However, this is consistent with the mutual coordination of cytosine methylation in the three sequence contexts, where CG, CHG, and CHH may play equally important roles in PWN (Figure S1).

### 3.3. Identification and Analysis of DMCs and DMRs

**Figure 3:**
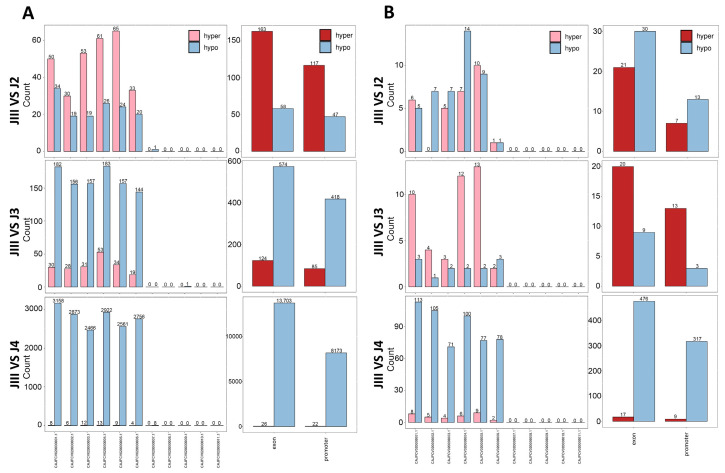
Genome-wide distribution of DMCs and DMRs throughout the four different developmental stages in pinewood nematode. (**A**) The number of hypermethylated and hypomethylated DMCs is counted and distributed across chromosomes and genetic elements in three pairwise comparisons: JIII vs. J2, JIII vs. J3, and JIII vs. J4. The distribution of the chromosome and the exons and promoters is shown in the left and right columns, respectively. (**B**) The number of hypermethylated and hypomethylated DMRs is counted and distributed across chromosomes and genetic elements.

To research genome-wide differences in DNA methylation during various developmental stages of PWN and to determine the role of DNA methylation in JIII formation, we conducted a differential methylation analysis of 33,446,454 CpG sites using 12 samples. We used the software MethylKit and Metilene to identify DMCs and DMRs between groups, respectively (see Methods for details), and mapped hypermethylated and hypomethylated differentially methylated sites and regions to chromosomes and genetic elements (Figure 3). Based on the distribution analysis of DMCs on chromosomes in pairwise comparisons among different groups, it can be observed that DMCs exhibit a coordinated distribution pattern across the six chromosomes of PWN (Figure 3A). This finding aligns with the overall trend of DNA methylation in the genome (Figure 2A and Figure 3A). By examining the status and trends of methylation of DMCs between pairwise comparisons (JIII vs. J2, JIII vs. J3, and JIII vs. J4), we observed a transition from a hypermethylated to a hypomethylated state in PWN from pre-JIII to post JIII (Figure 3A). In the pairwise comparison between JIII and J2, the counts of highly methylated differentially methylated cytosines (DMCs) were higher than the counts of low methylated DMCs on each chromosome. This trend was also observed in the status of exons and promoters. However, in the JIII vs. J3 pairwise comparison, the majority of DMCs were found to be hypomethylated. Similarly, in the JIII vs. J4 comparison, almost exclusively hypomethylated DMCs were counted (Figure 3A). Due to the low overall level of DNA methylation in PWN, the distribution of differentially methylated cytosines (DMCs) on chromosomes is sparse, we conducted further analysis and identified a total of 72, 57, and 578 differentially methylated regions (DMRs) in the three pairwise comparisons of JIII vs. J2, JIII vs. J3, and JIII vs. J4, respectively (Figure 3B).

### 3.4. GO Analysis of DMRs during JIII Formation

**Figure 4:**
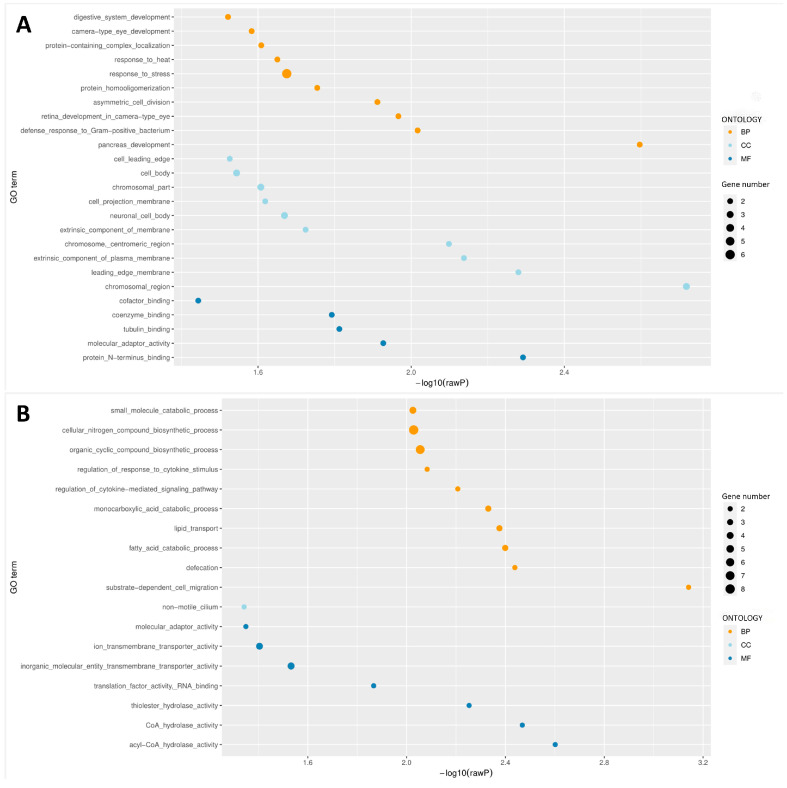
GO enrichment results of DMGs in DMRs. (**A**) Functional enrichment analysis of genes modified by hypermethylated DMRs in the experimental group relative to the control group (*Bursaphelenchus xylophilus* JIII vs. J2). (**B**) Enrichment of genes modified by hypomethylated DMRs in JIII vs. J2. The y-axis indicates the pathway name, x-axis indicates the enrichment factor, and the bubble size indicates the number of genes in each pathway. The figure only displays entries with statistical significance (rawP < 0.05). If there are more than 10 significantly enriched pathways for each type, only the results of the top 10 pathways of each type will be shown. GO is divided into three ontologies, namely: Molecular Function (MF), Cellular Component (CC), and Biological Process (BP).

To understand the formation of JIII, J2 is considered a necessary stage. Therefore, our study primarily focused on the functional enrichment and subsequent transcriptional analysis of JIII compared to J2. We performed Gene Ontology (GO) analysis for genes located in the DMRs with a significance threshold set at 0.05 (Table S4). Differentially methylated genes (DMGs) in hypermethylated DMRs were significantly enriched in 35 GO entries, including 15 biological process (BP) entries, 15 cellular component (CC) entries, and 5 molecular function (MF) entries (Table S5 and Figure 4A). The main entries enriched in BP include pancreas development (GO:0031016), defense response to Gram-positive bacterium (GO:0050830), retina development in the camera-type eye (GO:0060041), asymmetric cell division (GO:0008356), protein homooligomerization (GO:0051260), response to stress (GO:0006950), response to heat (GO:0009408) and other adaptation processes, whereas entries significantly enriched in CC mainly include chromosomal region (GO:0098687), leading-edge membrane (GO:0031256), an extrinsic component of the plasma membrane (GO:0019897), chromosome, centromeric region (GO:0000775), extrinsic component of membrane (GO:0019898), neuronal cell body (GO:0043025) and other components, in MF include protein N-terminus binding (GO:0047485), molecular adaptor activity (GO:0060090), tubulin binding (GO:0015631), coenzyme binding (GO:0050662), and cofactor binding (GO:0048037) (Figure 4A).

DMGs in hypomethylated DMRs were significantly enriched in 71 GO entries, including 63 BP entries, 1 CC entry, and 7 MF entries (Table S6 and Figure 4B). The hypermethylated DMGs are highly involved in BP entries including substrate-dependent cell migration (GO:0006929), defecation (GO:0030421), fatty acid catabolic process (GO:0009062), lipid transport (GO:0006869), monocarboxylic acid catabolic process (GO:0072329) and other processes of substance synthesis and metabolism. Unlike hypermethylated DMGs, only non-motile cilium (GO:0097730) was involved in the CC entries, acyl-CoA hydrolase activity (GO:0047617), CoA hydrolase activity (GO:0016289), thiolester hydrolase activity (GO:0016790), and other active functions in MF entries were involved in hypomethylated DMGs (Figure 4B).

### 3.5. Summary of Transcriptome Analysis and Pathway Enrichment

**Figure 5:**
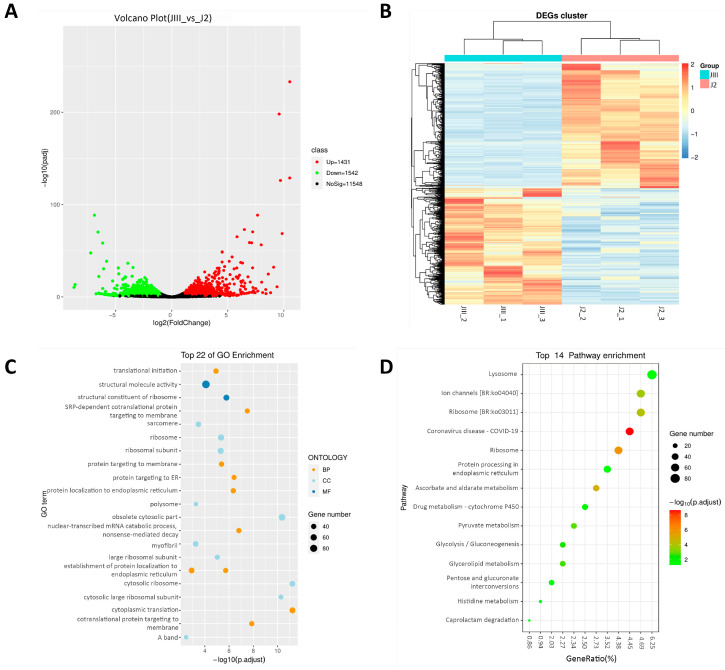
RNA sequencing analysis during *Bursaphelenchus* xylophilus JIII formation. (**A**) Volcano plot of differentially expressed genes (DEGs) in JIII vs. J2. The red and green dots indicate significantly up- and down-regulated differential genes, and black dots indicate non-significant differential genes. (**B**) Heatmap of DEGs in JIII vs. J2. Red indicates the upregulated genes, and purple indicates the downregulated genes. (**C**) GO enrichment of DEGs in JIII vs. J2. GO is divided into three ontologies, namely: Molecular Function (MF), Cellular Component (CC), and Biological Process (BP). (**D**) KEGG pathway enrichment of DEGs in JIII vs. J2. The size of the point represents the total number of genes enriched to the entry, with smaller p.adjust values indicated by a more red color.

To investigate the potential relationship between methylation changes and transcriptional patterns during JIII formation, we initially conducted transcriptome sequencing on the tested PWN. After raw data filtering, each sample contained an average of 22.91 M clean reads with 6.16 Gb (>Q30) (94.42%) (Table S7). After comparing the clean reads to the reference genome, we observed an average mapping rate of 92.21% per sample (Table S7). This indicates that the data has a relatively high matching rate. This analysis revealed the identification of 2973 differentially expressed genes (DEGs) during JIII formation. Among these DEGs, 1542 were downregulated and 1431 were upregulated (Table S8 and Figure 5A). For the 2973 DEGs identified in the JIII vs. J2 group, the cluster analysis revealed distinct results. The cluster plot displayed the samples from the JIII group and the J2 group clustering together, indicating noticeable disparities in gene expression patterns between the two groups (Figure 5B). This suggests significant alterations in biological processes, signaling pathways, or regulatory mechanisms between the JIII and the J2.

GO enrichment analysis and KEGG pathway analysis were further conducted on DEGs to gain insights into their biological significance and potential functions (Figure 5C,D). The GO enrichment terms were also classified by MF, CC, and BP terms (Figure 5C). To further explore the biochemical metabolic pathways and signal transduction pathways involved in the formation of JIII, we conducted a KEGG analysis using the KEGG pathway database. This analysis allowed us to annotate a total of 1995 DEGs into 379 pathways (Figure 5D). The pathways included coronavirus disease—COVID-19, ribosome, ascorbate and aldarate metabolism, ion channels [BR: ko04040], ribosome [BR: ko03011], pyruvate metabolism, and glycolysis/gluconeogenesis. By analyzing the DEGs within these pathways, we can gain further insights into the specific genes and molecular mechanisms that contribute to JIII formation.

### 3.6. Correlation Analysis of DMRs and Gene Expression during JIII Formation

**Figure 6:**
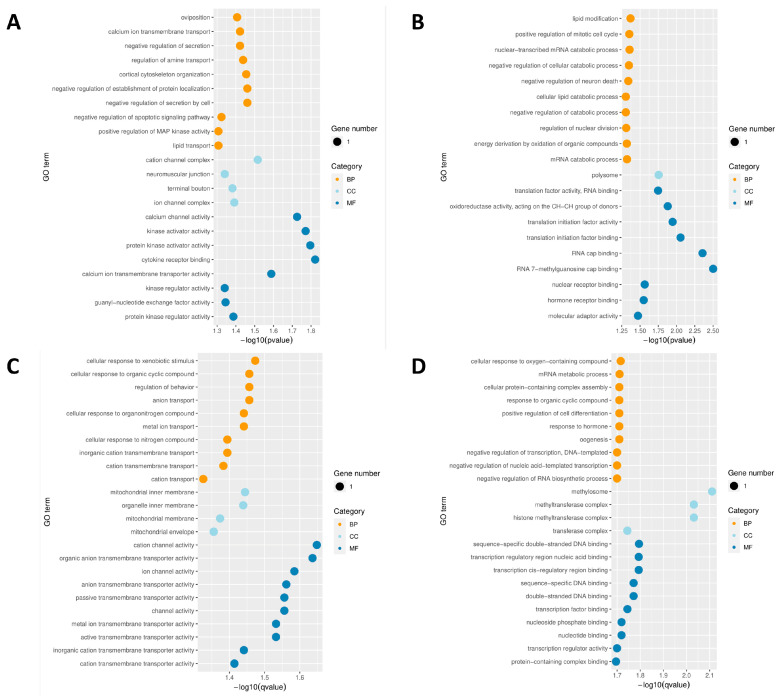
GO enrichment of genes with hypomethylation and transcriptional alterations in *Bursaphelenchus xylophilus* JIII vs. J2. (**A**) GO enrichment for genes that are methylated in genebody while transcription is decreased. (**B**) GO enrichment for genes that are methylated in genebody while transcription is elevated. (**C**) GO enrichment for genes that are methylated in promoter while transcription is decreased. (**D**) GO enrichment for genes that are methylated in promoter while transcription is elevated.

To examine the correlation between methylation changes in PWN and transcriptional patterns, we conducted a comparative analysis of DMRs and DEGs. Specifically, we performed a Venn analysis to identify common genes among the DMRs annotated in the CG sites of the promoter region and genebody region, as well as the DEGs. This analysis revealed a total of 23 genes that exhibited significant up-regulation or down-regulation in both the DMRs and DEGs datasets (Table 1). Compared to J2, JIII exhibited 6 hypermethylated genes in the promoter region, 3 hypomethylated genes in the promoter, 7 hypermethylated genes in the genebody, and 7 hypomethylated genes in the genebody (Table 1). Among the aforementioned hypermethylated genes, the majority did not show enrichment in related pathways. However, it is worth noting that most of the hypomethylated genes in the list exhibited significant enrichment in GO terms (Figure 6). This suggests that the process of hypomethylation may play a pivotal role in the formation of JIII by modulating gene expression. In the GO enrichment analysis, it was observed that the majority of enriched terms were associated with ion and compound transport, metabolic processes, transcription, and translation, as well as signal transduction and interactions with signal molecules. Notably, among the hypomethylated genes in the genebody, there was enrichment in lipid transport, lipid modification, and cellular lipid catabolic process (Figure 6A,B). Additionally, among the up-regulated genes, the KEGG pathway (Table S9) analysis revealed enrichment in fatty acid elongation and fatty acid biosynthesis. Our data further reveal that during the formation of JIII, two genes (*Enr* and *Pex10*) involved in fatty acid synthesis and maintenance of lipid balance undergo methylation changes within the gene body and the CpG island of the promoter, respectively.

### 3.7. Validation of DNA Methylation and Transcriptomes

**Figure 7:**
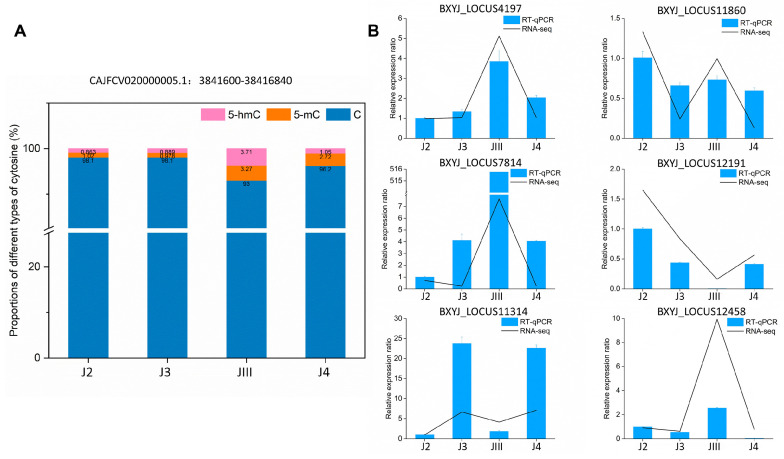
Validation of RNA-seq and DNA methylation using RT-qPCR. (**A**) DNA methylation verification of selected DNA fragment. (**B**) RT-qPCR validation in RNA-Seq results.

To further validate the accuracy of the methylation levels obtained from whole-genome bisulfite sequencing (WGBS), we utilized a methylation quantification kit (see the Methods Section for details) to select three DNA fragments containing hydroxymethylcytosine and methylated cytosine restriction sites. The methylation status of these fragments was then confirmed through RT-qPCR. The results indicated that two of the DNA fragments (ΔCq ≤ 0.5) exhibited no detectable methylation modifications, while one fragment showed a low level of dual methylation modifications (Figure 7A). Overall, the methylation levels determined through RT-qPCR were in good agreement with the sequencing results, further supporting the reliability of the methylation data obtained from WGBS. To validate the reliability of the RNA sequencing data, we randomly selected six genes that were identified in the sequencing analysis. The results obtained from RT-qPCR showed a high degree of consistency with the overall trend of gene expression levels observed in the RNA sequencing data (Figure 7B). This further confirms the accuracy and reliability of the RNA sequencing results.

## 4. Discussion

Epigenetic modifications do not alter the genome sequence itself, but they can lead to variations in biological phenotypes [40,41,42]. DNA methylation, as a crucial epigenetic modification, has been increasingly recognized for its role in regulating various biological processes. It has been found that DNA methylation can confer stability and heritability to certain phenotypic traits across generations, such as environmental adaptation and rapid response to unexpected adverse conditions [19,41]. With the rapid spread of PWN in China, it is crucial to understand the mechanisms underlying its invasion, expansion, and environmental adaptability. Epigenetic modifications play a significant role in facilitating the rapid response of PWN to environmental changes and have a profound impact on its invasion and adaptation. Investigating the molecular mechanisms that drive PWN expansion will provide valuable insights and contribute to a comprehensive understanding of this process. Therefore, constructing a comprehensive genome-wide methylation map of PWN will be instrumental in unraveling the methylation regulation information about crucial biological processes during JIII formation. In this study, we carefully selected four distinct developmental stages of PWN to generate the first set of match-to-genome DNA methylation data. To the best of our knowledge, this is the first report documenting the changes in DNA methylation patterns specifically during the formation of JIII.

We initially compared the overall level and genome distribution of DNA methylation among the four different developmental stages (J2, J3, JIII, and J4) of PWN. It was observed that DNA methylation exhibited a consistently low and stable level, with sparse distribution across the genome. This finding aligns with the trend observed in other species, where methylation patterns tend to remain stable throughout the lifespan. The relative stability of methylation patterns is a common feature in many eukaryotes [24]. In comparison to *Trichinella spiralis*, a parasitic nematode, PWN exhibits a lower overall level of methylation [28]. The higher methylation observed in JIII compared to other stages may be a response to changes in the external environment. Numerous studies have demonstrated that the level of DNA methylation within the same species can vary significantly at different developmental stages or under specific environmental conditions. For instance, the methylation level of mycelium in *Beauveria bassiana* is higher than that of conidia, as fungi produce dormant spores capable of surviving unfavorable conditions [43]. Additionally, the methylation level of zebrafish increases during short-term cold stress and decreases after long-term cold stress, indicating a potential role of methylation in regulating such responses [19]. These findings suggest that DNA methylation is involved in the organismal adaptation to environmental changes. In contrast to many other species, the level of DNA methylation in PWN remains stable across different developmental stages. Moreover, the overall increase in methylation during the JIII stage exhibits exceptional stress resistance. This phenomenon may be attributed to the loss of certain methyltransferases in PWN during the evolution of nematodes [22], although further research is required to confirm this.

We conducted a differential methylation analysis comparing JIII with three other developmental stages of PWN and observed significant changes in DNA methylation across the genome. During JIII formation, the majority of DMC showed hypermethylation. In contrast, when compared with J3 and J4 stages in the propagative cycle, most of the DMC exhibited hypomethylation. These findings suggest that DNA methylation in PWN may be rapidly sensed and adjusted during the process of JIII formation, particularly in response to adverse external environments. The relationship between DNA methylation and gene expression is complex, but generally, hypermethylation is associated with decreased transcription levels [44]. Additionally, hyper-DMCs may contribute to the stabilization of heterochromatin formation by suppressing the expression of relevant genes, thereby promoting the longevity of organisms [45]. JIII can survive for extended periods under low-temperature conditions, which may be attributed to the presence of hypermethylated DMCs. Due to the sparse distribution of methylation in the *B. xylophilus* genome, the number of DMRs identified was relatively small. However, during JIII formation, there was a higher proportion of hypomethylated DMRs. Studies have demonstrated that related genes exhibit a trend of low methylation and high expression, which is considered to be an adaptive strategy for organisms to thrive in wild environments [46]. This trend towards hypomethylation in gene bodies may reflect the transcriptional activity of associated genes, potentially increasing viability in JIII.

The second main focus of this study was to investigate the relationship between methylation changes and transcription during JIII formation. By overlaying DMRs and DEGs, a total of 23 genes were identified, showing changes in both methylation and gene expression. Functional analysis of these genes revealed that among the genes that were hypomethylated within the gene body, those that were upregulated in gene expression were significantly enriched in processes related to lipid transport, lipid modification, fatty acid elongation, and fatty acid synthesis. Previous studies have demonstrated that during JIII formation in PWN, there is a significant increase in fatty acid content and the proportion of unsaturated fatty acids [47]. Additionally, glycerol levels are significantly higher, while glucose levels are significantly lower compared to the propagative stage of the PWN [47]. These results suggest that the hypomethylated genes involved in lipid-related processes and the up-regulated genes associated with fatty acid metabolism may play crucial roles in JIII formation. The enrichment of lipid-related pathways and processes indicates the importance of lipid metabolism and transport in the development and maturation of JIII.

During the formation of JIII, we found that *Enr* and *Pex10* undergo methylation changes within the CpG island of the genebody and promoter, respectively. Importantly, there exists an inverse relationship between methylation and transcription of these two genes, with hypomethylation accompanied by increased transcription. This observation suggests a methylation-driven transcription mechanism, indicating that JIII formation involves targeted methylation at specific sites that control gene expression in response to adverse environmental conditions. The changes in methylation and transcription of *Enr* and *Pex10* can account for the alterations in lipid droplet abundance observed in JIII. As a crucial component of cellular metabolic pathways, fatty acids are essential for biological survival [48]. The *Enr* gene plays a significant role in the aggregation of lipid droplets during JIII formation and survival in adverse environments. *Enr* serves as the primary enoyl carrier protein reductase for type II bacterial fatty acid synthesis and is indispensable for the intracellular growth of *Listeria monocytogenes* [49]. The peroxisomal membrane protein (*Pex10*) functions as a RING finger E3 ligase that ubiquitinates PEX5, initiating its recycling into the cytoplasm or degradation [50]. Peroxisomes are organelles present in almost all eukaryotic cells and play a crucial role in lipid metabolism processes [51]. Extensive research has shown that peroxisomes and lipid droplets are physically associated with cells and closely connected [52]. Deletion of the *Pex10* gene in *Botrytis cinerea* leads to significant reductions in melanin production, fatty acid utilization, hyperosmolarity, and tolerance to reactive oxygen species (ROS) [53]. JIII is strongly associated with lipid metabolism, highlighting the relevance of the *Pex10* gene in lipid metabolism and stress resistance. These findings provide valuable insights into the molecular mechanisms underlying the unique characteristics of JIII and highlight the significance of lipid metabolism in the life cycle of PWN.

## 5. Conclusions

Collectively, our study provides the first genome-wide distribution of DNA methylation levels in PWN, confirming the presence of DNA methylation across four developmental stages of the nematode. We observed a significant difference in methylation between the dispersive JIII and the propagative stage of PWN, suggesting its potential role in JIII’s resistance to adverse environmental conditions. Our findings also support the involvement of DNA methylation in the regulation of related genes during JIII formation. Highly expressed genes in the hypomethylated regions may contribute to JIII formation and environmental adaptability through the regulation of lipid metabolism pathways. Furthermore, in the process of JIII formation, the down-regulation of methylation levels in *Enr* and *Pex10* genes can promote the expression of these genes. This, in turn, enhances the involvement of JIII in fatty acid synthesis and lipid balance maintenance, enabling it to survive in adverse environments. This study offers novel insights into the rapid expansion mechanism of PWN and establishes a theoretical foundation for the prevention and control of pine wilt disease. However, further studies are needed to elucidate the regulatory mechanisms of DNA methylation.

## Figures and Tables

**Table 1:** Genes with altered methylation and transcription in *Bursaphelenchus xylophilus* JIII vs. J2. The gene IDs listed are the transcript names in the reference genome. For genes with documented homologs (gene name in parentheses), transcript functions listed in NCBI were used to determine putative identity.

Gene ID	DMR Genomic Location	DMR Difference	DEG log2 Fold Change
BXYJ_LOCUS13082	genebody	0.338333	−1.104336548
BXYJ_LOCUS11405 (Syt1)	genebody	0.200167	−1.268410936
BXYJ_LOCUS7569	genebody	0.184889	6.037514936
BXYJ_LOCUS7569	promoter	0.184889	6.037514936
BXYJ_LOCUS7568	promoter	0.184889	6.477511571
BXYJ_LOCUS8847	genebody	0.148556	−1.069283441
BXYJ_LOCUS12505	promoter	0.128333	1.891287088
BXYJ_LOCUS15791 (Kmt)	genebody	0.119778	1.718757485
BXYJ_LOCUS10978	genebody	0.1175	−1.451330359
BXYJ_LOCUS8383	genebody	0.117333	7.439557963
BXYJ_LOCUS8383	promoter	0.117333	7.439557963
BXYJ_LOCUS8384	promoter	0.117333	3.671438732
BXYJ_LOCUS10824	promoter	0.102778	−3.842519089
BXYJ_LOCUS8048 (Trpv6)	genebody	−0.100111	−1.312559644
BXYJ_LOCUS10582 (Enr)	genebody	−0.100778	2.43271744
BXYJ_LOCUS6456 (Pex10)	promoter	−0.132444	1.010721634
BXYJ_LOCUS8233 (Col-125)	genebody	−0.15	−4.15283697
BXYJ_LOCUS7390 (Kcnk18)	promoter	−0.161667	−1.063657134
BXYJ_LOCUS12977 (Srt-41)	genebody	−0.166333	−1.476196334
BXYJ_LOCUS5952 (Madd)	genebody	−0.171556	−1.15646022
BXYJ_LOCUS3255 (Nht-62)	genebody	−0.219333	−1.199834112
BXYJ_LOCUS12921 (Slc25a10)	promoter	−0.227556	−1.178271087
BXYJ_LOCUS5381 (Mif4gd)	genebody	−0.244333	1.390619044

## Data Availability

The datasets presented in this study can be found in online repositories. The names of the repository and accession number can be found below: https://www.ncbi.nlm.nih.gov/geo/ (accessed on 1 November 2023), GSE242183.

## Supplementary Materials

The following supporting information can be downloaded at: https://www.mdpi.com/article/10.3390/cimb45120603/s1, Table S1: location information and primer sequences of validated RNA-seq genes and methylated DNA fragments; Table S2: summary of whole-genome bisulfite sequencing data from 12 samples; Table S3: average methylation levels in 12 samples; Table S4: differentially methylated genes (DMGs) in JIII vs. J2; Table S5: GO analysis of hypermethylated differentially methylated genes (DMGs) in JIII vs. J2; Table S6: GO analysis of hypomethylated differentially methylated genes (DMGs) in JIII vs. J2; Table S7: summary of RNA-seq data from 12 samples; Table S8: differentially expressed genes (DEGs) in JIII vs. J2; Table S9: KEGG pathway for hypomethylated differentially methylated genes (DMGs) in genebody while transcription is elevated in JIII vs. J2; Figure S1: the distribution of methylation levels in the four different stages of *B. xylophilus*.
