# Supplementary material for: Analysis of DNA Methylation Differences during the JIII Formation of Bursaphelenchus xylophilus
Source: Curr Issues Mol Biol. 2023 Nov 30;45(12):9656–73. doi: 10.3390/cimb45120603 (PMC10742416; doi:10.3390/cimb45120603)
Supplement: Supplementary file 1 [file cimb-45-00603-s001.zip › Supplementary Figure.pdf]

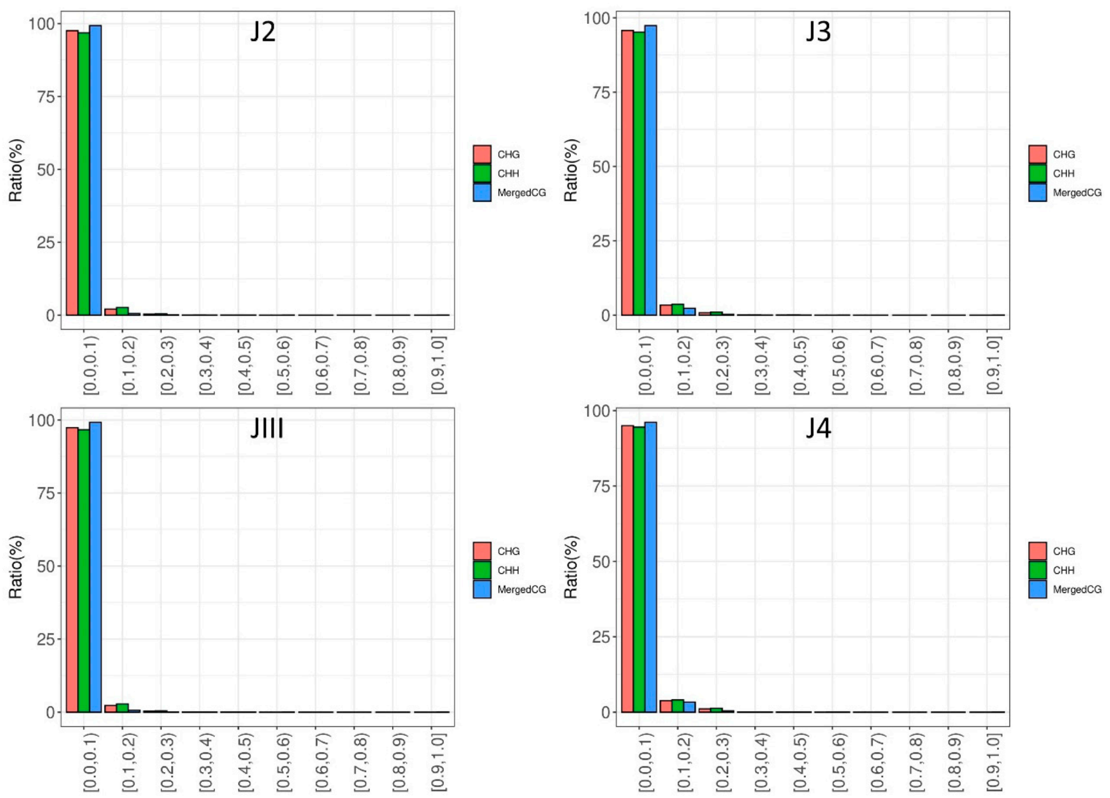

Figure S1. The distribution of methylation levels in the four different stages of *B. xylophilus*. The distribution is presented as a histogram, divided into 10 levels according to methylation level (0-0.1, 0.1-0.2, 0.2-0.3, etc).
